# Environmental and social determinants of acute rheumatic fever: a longitudinal cohort study

**DOI:** 10.1017/S0950268818003527

**Published:** 2019-01-28

**Authors:** J. W. Cannon, M. Abouzeid, N. de Klerk, C. Dibben, J. R. Carapetis, J. M. Katzenellenbogen

**Affiliations:** 1 Wesfarmers Centre for Vaccines and Infectious Diseases, Telethon Kids Institute, University of Western Australia, Subiaco, WA, Australia; 2 Centre for Humanitarian Leadership, A Save the Children Australia – Deakin University Partnership, Burwood, VIC, Australia; 3 Telethon Kids Institute, University of Western Australia, Subiaco, WA, Australia; 4 School of Geosciences, University of Edinburgh, Edinburgh, Scotland; 5 School of Population and Global Health, University of Western Australia, Crawley, WA, Australia

**Keywords:** Epidemiology, rheumatic fever, rheumatic heart disease, *Streptococcus pyogenes*

## Abstract

Acute rheumatic fever (ARF), an auto-immune response to a group A *Streptococcus* infection and precursor to rheumatic heart disease (RHD), remains endemic in many socio-economically disadvantaged settings. A Global Resolution on ARF and RHD was recently adopted at the 71st World Health Assembly where governments committed to improving efforts to prevent and control ARF and RHD. To inform these efforts, the objectives of this study were to examine associations between childhood ARF in the UK between 1958 and 1969 and a range of environmental and social factors. Of 17 416 children from the nationally representative birth cohort of the National Child Development Study, ARF was reported in 23 children during early childhood (between birth and the 7-year follow-up) and in 29 additional children during middle childhood (between the 7- and 11-year follow-ups). Risk factors associated with ARF in both early and middle childhood were: a large family size; attendance at a private nursery or class; a history of nephritis, kidney or urinary tract infections; and a history of throat or ear infections. Risk factors for ARF in early childhood alone were families with fathers in a professional or semi-professional occupation and families who moved out of their local neighbourhood. Risk factors in late childhood alone included overcrowding and free school meals. These data suggest that prevention strategies in ARF endemic settings may be enhanced by targeting, for example, new members entering a community and children in environments of close contact, such as a nursery or shared bedrooms.

## Short repot

Acute rheumatic fever (ARF) occurs as a result of an abnormal immune response to a group A *Streptococcus* (GAS) infection [1], with children aged 5–14 years at greatest risk [2]. The incidence of ARF has declined during the latter part of the 20th century at a global level, demonstrating that it is entirely preventable. However, it was sufficiently high in England and Wales to warrant the initiation of compulsory case notification from late 1947, which demonstrated an incidence rate ranging between 25 and 55 cases per 100 000 children aged <17 between 1948 and 1953 [3]. In the present era, ARF persists in socio-economically disadvantaged settings, predominately in low- and middle-income countries (LMICs) but also among Indigenous and other vulnerable populations in high-income countries [4]. This means that learnings from the UK in the early and mid-20th century have contemporary significance.

Moreover, ARF can lead to heart valve damage, known as rheumatic heart disease (RHD), which is estimated to affect over 33 million people globally and result in approximately 320 000 deaths annually [5]. Because such a high burden of disease persists in disadvantaged populations, a Global Resolution on ARF and RHD was recently adopted at the 71st World Health Assembly (25 May 2018); unanimously adopted by Member States, the resolution calls for improved efforts to prevent and control ARF and RHD.

Given the persistence of ARF and RHD in socio-economically disadvantaged settings, it stands to reason that an association exists between ARF and a range of environmental and social determinants of health. However, despite an assumed relationship between social determinants of health, the evidence remains weak. A recent systematic review identified a paucity of quality studies investigating these associations, with over half of the papers reviewed being assessed as very poor to poor quality [6]; however, although the populations and settings examined, the parameters analysed, and the strength and direction of associations reported in the studies varied, a causal relationship with crowding and with socio-economic status was suggested [6]. Thus, further studies are required to help clarify the relationship between ARF and environmental and social factors.

In this short report, we examine the associations between childhood ARF and a range of environmental and social factors, using data from the 1958 National Child Development Study (NCDS). Evidence of these associations is central to efficient prevention strategies that aim to reduce the endemic burden of disease in resource-limited settings of LMICs and other disadvantaged communities.

The 1958 NCDS is a unique nationally representative cohort study that followed 17 416 people who were born in England, Scotland and Wales in a single week of March 1958, and immigrants born in the same week who were living in Britain during data collection periods up to age 16 years [7]. Longitudinal follow-up of this cohort has occurred at birth and at ages 7, 11, 16, 23, 33, 41/42, 46/47, 50 and 55 years. The follow-ups have involved data collection by a range of methods and sources, including interviews of parents and the subject themselves, school teachers, medical examinations, spouses and their children in later waves, and linkage with census data. This current study examines data collected in the first three waves (ages 0, 7 and 11 years). Access to these data was provided by the UK Data Service (usage number 118 439).

The primary outcome for our analyses was a history of ARF as reported by the child's parent at the 7- or 11-year follow-ups (a history of ARF was not collected at age 16). Of the wide range of environmental and social exposures collected at the birth and the 7- and 11-year follow-ups by the NCDS, the plausible risk factors for ARF that were analysed in this study are shown in Table 1. This table gives a brief description of each risk factor along with the corresponding variable names given by the NCDS data custodians, which are available to researchers accessing these data; for example, paternal socio-economic group according to occupation was collected at birth and the 7- and 11-year follow-ups with the variable names n236, n190 and n1687, respectively. Additionally, a composite variable of family size and overcrowding (>1.5 persons per room) was derived with the three categories of (1) households that were not overcrowded and with family size at or below the cohort median (reference category), (2) households that were not overcrowded but with a larger family size and (3) households that were overcrowded regardless of family size. The variable and definition of overcrowding was defined previously by Elliott and Lawrence [8], which is consistent with the United States Census Bureau that classifies >1.5 persons per room as ‘severely overcrowded’.

**Table 1.** Risk factors for acute rheumatic fever stratified by statistical significance (*P* < 0.1)

Description of variables	NCDS 1958 variable name	
	No association	Significant association
Socio-economic (SE)		
SE group according to occupation		
Paternal		n236*, n190*, n1687*
Father's father	n193	
Mother's father	n526, n660	
Parent educational ascertainment	n537, n194	
Mother employed	n539, n540, n147, n197, n212	
Residential region	n0region, n1region, n2region	
Free school meals	n1229	n858
Household		
Number of persons (birth, 7-year)		n546*, n419*, n99*
Persons per room (birth, 7-year)		n512*, n607*
Number of persons (11-year)	n1116*, n1117* (under 21)	
Persons per room (11-year)	n1683	
Child shares bedroom/bed	n1157/8	
Child's birth order (e.g. first born)	n101, n1118	
Type of accommodation	n199	
Tenure of accommodation	n200, n1152	
Amenities	n204-208, n621, n1159-63, n1681	
Community		
Move out of local area		n97*
Number of house moves	n95, n1150	
Attended nursery school or class	n105 (LA, excl. day), n107 (LA, day), n108 (other activity or pvt day)	n106 (pvt, excl day)
Play group	n122, n941	
Number schools attended	n112, n1135	
Number of pupils in school and class	n24, n46, n828, n865	
Child in ‘care’ or institution	n132, n1113, n1133	
Health		
Scarlet fever	n220	
Tonsillitis or tonsils removed	n246, n1353	
Recurrent throat or ear infections	n1348 (11-year)	n256 (7-year)
Nephritis, kidney or other U-G tract		n285
Skin conditions	n270-1, n414, n1487	
Poor appetite	n130	
Other		
Mother's age at child's birth	n553	
Father's age at child's birth		n494
Parents’ country of birth	n1434*, n1436*	
Gestational period	n497	
Birth weight	n574, n646, n515-6	
Breast fed		n222*
Sucks thumb/finger	n141	

LA, local area; pvt, private.

National Child Development Survey (NCDS) variable names are as recoded by the Centre for Longitudinal Studies, UK.

*Recoded variable.

Statistical comparisons of ARF and non-ARF cases were made using *t*-tests, *χ*^2^ tests or Fisher's exact test where appropriate. Associations with a *P*-value <0.1 were considered significant given the low number of cases with ARF, the results of which are shown in the stratification by significance in Table 1. This higher than traditional cut-off value was used because of the low number of ARF cases; however, it increases the likelihood of finding a significant association when one does not exist (type I error).

We further analysed the association between the risk factors found to be significant and ARF occurring in early childhood (between birth and the 7-year follow-up) and middle childhood (between the 7- and 11-year follow-ups). ARF was stratified by age at onset because there is increasing evidence that ARF may be caused by GAS impetigo, which can occur at an earlier age than GAS pharyngitis and reflect different risk factors, and some variables were not collected consistently across each follow-up. Univariate analyses of significant associations were conducted with Firth's logistic regression, reporting odds ratios (ORs) and 95% confidence intervals (95% CI). Multivariable analyses were not conducted given the small number of ARF cases. However, the composite family size/overcrowding variable tested if a large family size was independent of overcrowded environments in the risk of ARF.

All analyses were performed using R: A Language and Environment for Statistical Computing (version 3.4.0, R Foundation for Statistical Computing, Vienna, Austria); Firth's logistic regression was performed using the package logistf: Firth's Bias-Reduced Logistic Regression (version 1.22). An approval for exemption from ethics review was obtained from the University of Western Australia (RA/4/20/4616) and Deakin University (2018-214) on the grounds that this analysis used pre-existing non-identifiable data.

The primary outcome of ARF was reported in 59 children from at least one of the 7- and 11-year follow-ups (15 997 valid responses). This total comprised 23 cases in early childhood (of 14 545 valid responses to the 7-year follow-up); 29 new cases in middle childhood (of 13 292 valid responses to both the 7- and 11-year follow-ups, crude incidence rate of 55 per 100 000 person-years); and seven cases reported at the 11-year follow-up but not responding to the 7-year follow-up.

The mean (standard deviation) ages of the mother and father at the time of the child's birth were 27.5 (5.7) and 30.6 (6.4) years, respectively. Children who experienced an episode of ARF in early childhood were more likely to have an older father compared with those who did not (mean difference 3.1 years, 95% CI 0.3–6.0; *P* = 0.032).

The risk factors that had a statistically significant association with ARF developing in early or middle childhood are shown in Table 2, and the results are described in this and the next three paragraphs. A higher family socio-economic status (SES) based on the father's occupation was associated with an increased risk of ARF in early childhood. There was no evidence that the same association with SES existed for ARF developing in middle childhood; however, and in contrast, there was an association with children who received free school meals.

**Table 2.** Univariate associations between acute rheumatic fever (ARF) and selected risk factors

	ARF ages 0–7 years	ARF ages 7–11 years
Risk factors	OR (95% CI)	OR (95% CI)
Socio-economic		
Paternal occupation at birth		
Non-professional	ref.	ref.
Prof/semi-prof	2.48 (0.90–6.21)*	0.93 (0.29–2.34)
Unknown/no male head	1.93 (0.21–7.98)	1.80 (0.36–5.56)
Paternal occupation at the 7-year follow-up		
Non-professional	ref.	ref.
Prof/semi-prof	3.12 (1.36–6.97)**	0.80 (0.25–2.01)
Unknown	3.13 (0.02–23.73)	1.09 (0.12–4.23)
No father/male head	0.99 (0.01–7.48)	0.80 (0.25–2.01)
Paternal occupation at the 11-year follow-up		
Non-professional	Not applicable	ref.
Prof/semi-prof		1.11 (0.42–2.58)
Unknown		2.86 (0.32–11.32)
No father/male head		3.46 (1.07–8.93)**
Free school meals (school survey)	Not applicable	3.54 (1.41–8.05)**
Household		
At birth		
No. of people (>median^a^)	2.15 (0.89–5.28)*	2.23 (1.07–4.69)**
Overcrowded (>1.5 persons per room)	0.50 (0.06–1.99)	2.72 (1.15–5.90)**
At the 7-year follow-up		
No. of people (>median^b^)	1.41 (0.60–3.16)	2.13 (1.02–4.46)**
Overcrowded (>1.5 persons per room)	0.39 (0.04–1.51)	2.16 (0.87–4.80)*
Community (all reported at the 7-year follow-up)		
Move since birth	0.71 (0.32–1.64)	0.57 (0.27–1.19)
Move since birth – out of local area^c^	2.39 (1.04–5.35)**	0.57 (0.18–1.41)
Attended private nursery/class (excl. day nursery)	5.16 (1.79–12.70)**	3.18 (1.01–7.94)**
Health/other (all reported at the 7-year follow-up)		
History nephritis, kidney or urinary tract infection	12.41 (3.27–34.74)**	6.08 (1.23–18.67)**
History >3 throat or ear infections with fever	3.15 (1.25–7.23)**	1.53 (0.54–3.59)
Breast fed as a baby	1.25 (0.53–3.33)	0.52 (0.25–1.10)*

LA, local area.

*P*-value less than *0.1 and **0.05.

a Cohort median at birth was 3 people.

b Cohort median at the 7-year follow-up was five people.

c Compared to families who did not move or moved within their local area.

Within the household, a large family size had a higher risk of ARF occurring in both early and middle childhood compared with a family of median size or less. When combining all cases who developed ARF between birth and the 11-year follow-up, the risk of ARF was substantially lower in single compared with multiple child households where there were no cases of ARF reported in single-child households (OR 0.1, 95% CI 0.0–0.7). Although a large family increased the risk of ARF, overcrowding was found to be a significant risk factor for ARF developing in middle childhood but not early childhood. Based on our composite variable, households that were not overcrowded but had a large family size, at both birth and the 7-year follow-up, were associated with ARF developing in early childhood (OR 3.3, 95% CI 1.3–9.0); however, this association was not significant for ARF developing in middle childhood.

Of the community factors, children who had moved out of their local neighbourhood or who had attended a private nursery or class had a higher risk of ARF in early childhood compared with those who had not. Attending a private nursery or class was also a risk factor for ARF in middle childhood. In contrast, there was no evidence that children who had attended a local area nursery were at increased risk of ARF at any age.

Several health-related factors were associated with ARF, namely a history of nephritis, kidney or urinary tract infections; a history of >3 throat or ear infections within the year preceding the 7-year follow-up; and not being breastfed as a baby.

This analysis is the first to utilise the rich environmental and social information collected in the NCDS to examine associations with ARF and potentially RHD. The direction of association with these factors differed for ARF occurring in early and middle childhood. The occurrence of ARF in early childhood was associated with factors consistent with a higher socio-economic status while the opposite was true for ARF occurring in middle childhood. Nonetheless, the crude incidence rate of 55 per 100 000 person-years in middle childhood reflects that ARF remained a health challenge in the UK in the 1960s.

The association between a higher skilled occupation of the child's father and ARF in early childhood was unexpected but does not necessarily contradict other studies. Of three studies analysing this association, none demonstrated statistical significance [9–11]. Assuming a professional or semi-professional occupation is an indicator of higher social-economic status, one explanation for this association may be that these families had better access to healthcare and, therefore, a greater chance of diagnosis.

Alternatively, a higher occupational level may have led to household moves out of the local area, or *vice versa*, and this factor, along with attendance at a private nursery or class during the same period, was associated with ARF in early childhood, but lacking information on the socio-economic trajectory of the household moves makes these suggestions fairly speculative. These associations have never been reported directly; however, other studies have demonstrated epidemics of pharyngitis and ARF in relatively closed populations due to new GAS strains introduced from outside sources [12, 13], and in military sites which are characterised by a periodic country-wide influx of new recruits and close living quarters [14, 15]. Thus, a change in residential area or increased close contact with other toddlers may have led to an increased exposure to new GAS strains or infections, and this finding suggests that similar factors could be targeted by primordial/primary prevention strategies in ARF endemic settings.

Increased exposure to GAS may also explain the associations between ARF and a large family size, which may also have resulted in overcrowding. In our study, overcrowding was not a necessary factor for ARF, as demonstrated by ARF in early childhood where only one case was from an overcrowded household, but we were unable to statistically demonstrate that a large family size was independent of overcrowding in the risk of ARF in middle childhood. These results suggest that both are important and focusing efforts on reducing overcrowding alone may not prevent ARF.

The strong association between nephritis, kidney or urinary tract infections could indicate high infection loads. Nephritis could also be an indicator for acute post-streptococcal glomerulonephritis, which is another major auto-immune response to GAS infection. However, it is difficult to draw any further conclusion from this result as all three conditions were combined into a single survey question.

A major strength of this study was the excellent population representation from the birth cohort design; the cohort included almost all births in England, Scotland and Wales in a single week of March 1958. The detailed information about socio-economic status and household environment also allowed a range of variables to be investigated. This NCDS is one of few such datasets globally that have collected extensive social, demographic, health and developmental information over the life-course and from which such insights may be gleaned. The major limitations were the retrospective and self-report ascertainment of ARF and other factors, with likely recall bias leading to an under-reporting of ARF. Importantly, the low number of cases ascertained that did not allow for an in-depth multivariable analysis to adjust for confounding.

These data suggest that, of the vast array of environmental and social conditions that are conducive to GAS infection, increased exposure and proximity to new peers may be the more important determinants of ARF. If so, primordial and primary prevention strategies in ARF endemic settings may be enhanced by targeting, for example, people who move a lot, new members entering a community and children in environments of close contact such as nursery or shared bedrooms.

This study has used a historical dataset to examine issues of considerable contemporary global policy relevance. The persistent burden of ARF/RHD in socio-economically deprived settings is an issue of global significance: in LMICs where RHD burden is rife, in developed nations where RHD persists among high-risk disadvantaged groups, and in humanitarian settings, where mass population displacement and poor living conditions render ARF/RHD an issue warranting consideration. The recently adopted World Health Assembly Resolution includes a call for Member States to take measures to address the underlying root causes of ARF and RHD, including poor housing and overcrowding. Building the evidence base around such primordial associations with ARF/RHD is therefore essential to inform contemporary policy action.

## Supplementary material

For supplementary material accompanying this paper visit https://doi.org/10.1017/S0950268818003527. click here to view supplementary material
