# Supplementary material for: Environmental and social determinants of acute rheumatic fever: a longitudinal cohort study
Source: Epidemiol Infect. 2019 Jan 28;147:e79. doi: 10.1017/S0950268818003527 (PMC6518546; doi:10.1017/S0950268818003527)
Supplement: Supplementary file 1 [file S0950268818003527sup001.docx]

**Appendix**

Table S1. Characteristics of the cohort at different follow-up periods stratified by history of acute rheumatic fever (ARF).

|  | ARF ages 0-7 years | | |  | ARF ages 7-11 years | | |
| --- | --- | --- | --- | --- | --- | --- | --- |
| Category of risk factors | No  N=14522 | Yes  N=23 | p-value |  | No  N=13263 | Yes  N=29 | p-value |
| **Socio-economic** |  |  |  |  |  |  |  |
| *At birth* |  |  |  |  |  |  |  |
| Non-professional | 11039 (78.6) | 12 (63.2) | 0.169 |  | 9895 (78.3) | 22 (78.6) | 0.815 |
| Prof/semi-prof | 2316 (16.5) | 6 (31.6) |  |  | 2128 (16.8) | 4 (14.3) |  |
| Unknown/no male head | 687 (4.9) | 1 (5.3) |  |  | 612 (4.8) | 2 (7.1) |  |
| *At the 7-year follow-up* |  |  |  |  |  |  |  |
| Non-professional | 11183 (77.0) | 13 (56.5) | 0.044 |  | 9163 (74.2) | 22 (75.9) | 0.51 |
| Prof/semi-prof | 2790 (19.2) | 10 (43.5) |  |  | 2297 (18.6) | 4 (13.8) |  |
| Unknown | 132 (0.9) | 0 (0.0) |  |  | 559 (4.5) | 1 (3.4) |  |
| No father/male head | 417 (2.9) | 0 (0.0) |  |  | 329 (2.7) | 2 (6.9) |  |
| *At the 11-year follow-up* |  |  |  |  |  |  |  |
| Non-professional | 8802 (65.7) | 8 (40.0) | 0.031 |  | 9369 (70.6) | 18 (62.1) | 0.157 |
| Prof/semi-prof | 2763 (20.6) | 8 (40.0) |  |  | 2970 (22.4) | 6 (20.7) |  |
| Unknown | 1241 (9.3) | 4 (20.0) |  |  | 265 (2.0) | 1 (3.4) |  |
| No father/male head | 594 (4.4) | 0 (0.0) |  |  | 659 (5.0) | 4 (13.8) |  |
|  |  |  |  |  |  |  |  |
| Free school meals (2S) | 1224 (9.9) | 1 (5.0) | 0.715 |  | 1220 (10.0) | 6 (23.1) | 0.026 |
|  |  |  |  |  |  |  |  |
| **Household** |  |  |  |  |  |  |  |
| *At birth* |  |  |  |  |  |  |  |
| Household size > median~ | 4707 (34.0) | 10 (52.6) | 0.086 |  | 4236 (34.0) | 15 (53.6) | 0.029 |
| Overcrowded (>1.5 persons per room) | 1893 (13.8) | 1 (5.3) | 0.350 |  | 1700 (13.8) | 8 (29.6) | 0.018 |
|  |  |  |  |  |  |  |  |
| *At the 7-year follow-up* |  |  |  |  |  |  |  |
| Household size > median^ | 4427 (31.7) | 9 (39.1) | 0.446 |  | 3665 (31.9) | 14 (50.0) | 0.040 |
| Overcrowded (>1.5 persons per room) | 2022 (14.6) | 1 (4.3) | 0.238 |  | 1652 (14.5) | 7 (25.9) | 0.091 |
|  |  |  |  |  |  |  |  |
| Only child | 1288 (8.9) | 0 (0.0) | 0.165 |  | 1000 (8.4) | 0 (0.0) | 0.166 |
|  |  |  |  |  |  |  |  |
| **Community interaction (at 7-year follow-up)** |  |  |  |  |  |  |  |
| Move since birth | 9187 (64.3) | 13 (56.5) | 0.437 |  | 7470 (63.8) | 14 (50.0) | 0.130 |
| Move since birth - out of local area† | 3544 (24.5) | 10 (43.5) | 0.035 |  | 2798 (23.6) | 4 (13.8) | 0.276 |
|  |  |  |  |  |  |  |  |
| LA nursery/class (excl. day nursery) | 1371 (9.7) | 1 (4.3) | 0.511 |  | 1093 (9.4) | 4 (14.3) | 0.513 |
| Private nursery/class (excl. day nursery) | 775 (5.7) | 5 (22.7) | 0.001 |  | 603 (5.5) | 4 (14.3) | 0.064 |
| LA day nursery | 409 (3.0) | 0 (0.0) | 0.653 |  | 314 (2.8) | 0 (0.0) | 0.636 |
| private day nursery/other group | 612 (4.3) | 2 (8.7) | 0.261 |  | 504 (4.3) | 3 (10.3) | 0.130 |
|  |  |  |  |  |  |  |  |
| **Health/other (at 7-year follow-up)** |  |  |  |  |  |  |  |
| Hx nephritis, kidney or urinary tract infection | 206 (1.4) | 3 (13.6) | 0.004 |  | 173 (1.5) | 2 (6.9) | 0.068 |
| Hx >3 throat or ear infections with fever | 1827 (12.6) | 7 (30.4) | 0.010 |  | 1508 (12.8) | 5 (17.2) | 0.473 |
| Breast fed | 9829 (68.3) | 17 (73.9) | 0.564 |  | 8097 (68.7) | 15 (53.6) | 0.085 |

LA, local area.

~cohort median at birth was 3 people; ^cohort median at the 7-year follow-up was 5 people; †compared to families who did not move or moved within their local area.
